# Splicing promotes the nuclear export of *β-globin* mRNA by overcoming nuclear retention elements

**DOI:** 10.1261/rna.051987.115

**Published:** 2015-11

**Authors:** Abdalla Akef, Eliza S. Lee, Alexander F. Palazzo

**Affiliations:** Department of Biochemistry, University of Toronto, Toronto, Ontario M5S 1A8, Canada

**Keywords:** mRNA nuclear export, mRNA nuclear retention, *β-globin*, UAP56, gene expression

## Abstract

Most current models of mRNA nuclear export in vertebrate cells assume that an mRNA must have specialized signals in order to be exported from the nucleus. Under such a scenario, mRNAs that lack these specialized signals would be shunted into a default pathway where they are retained in the nucleus and eventually degraded. These ideas were based on the selective use of model mRNA reporters. For example, it has been shown that splicing promotes the nuclear export of certain model mRNAs, such as human *β-globin*, and that in the absence of splicing, the cDNA-derived mRNA is retained in the nucleus and degraded. Here we provide evidence that *β-globin* mRNA contains an element that actively retains it in the nucleus and degrades it. Interestingly, this nuclear retention activity can be overcome by increasing the length of the mRNA or by splicing. Our results suggest that contrary to many current models, the default pathway for most intronless RNAs is to be exported from the nucleus, unless the RNA contains elements that actively promote its nuclear retention.

## INTRODUCTION

Eukaryotic cells contain two major compartments, the nucleoplasm where mRNA is synthesized and processed, and the cytoplasm where this mRNA is translated into proteins. It is currently believed that in vertebrate cells, mRNAs contain specialized *cis*-acting elements that recruit nuclear export factors and permit their efficient export to the cytoplasm (Palazzo and Akef 2012). This contrasts to the situation in *Saccharomyces cerevisiae* where nuclear export factors are recruited to the transcript during transcription, regardless of their sequence or the presence of any specialized *cis*-elements (Palazzo and Akef 2012).

Much of current thinking on this subject has been derived from studies of the main export complex in eukaryotic cells, the transcription export (TREX) complex. In vertebrate cells, it is believed that the TREX complex is primarily loaded onto the RNA in both a splicing and a cap-dependent manner (Zhou et al. 2000; Strässer and Hurt 2001; Masuda et al. 2005). TREX then recruits the heterodimeric nuclear transport receptor TAP/p15 (also known as NXF1/NXT1), which ferries the mRNA across the nuclear pore (Katahira et al. 1999; Stutz et al. 2000). Thus introns act as de facto export-promoting *cis*-elements and this has been validated by the observation that certain model mRNAs such as *fushi tarazu* (*ftz)* and *β-globin* (β*G*) are only exported when they contain introns and thus spliced (Luo and Reed 1999; Valencia et al. 2008). In contrast, yeast TREX components are loaded onto mRNAs cotranscriptionally by the action of RNA Pol II (Chávez et al. 2000; Jimeno et al. 2002; Strässer et al. 2002; Zenklusen et al. 2002). The lack of a splicing requirement in yeast makes sense in light of the fact that the vast majority of their protein-coding genes are intronless.

Within this context, it was assumed that human protein-coding genes that are naturally intronless would need some substitute *cis*-element to recruit the TREX complex in the absence of splicing. This led to the identification of putative cytoplasmic accumulation region-elements (CAR-Es) by the analysis of a handful of human intronless genes (Lei et al. 2011, 2012). This idea was validated by fusing 16 copies of the putative CAR-E consensus element to the 5′ end of the intronless β*G* mRNA (β*G*-Δ*i*) (Lei et al. 2012). Inexplicably, the mutation of putative CAR-Es from naturally intronless mRNAs did not affect the nuclear export (Lei et al. 2012), suggesting that the export of these mRNAs may not be dependent on these elements.

In parallel with these studies, several groups have mapped and identified *cis*-elements within long noncoding RNAs (lncRNAs) that promote their nuclear retention. Interestingly, when these elements are eliminated, the lncRNAs are typically exported to the cytoplasm even though they lack introns (Miyagawa et al. 2012; Zhang et al. 2014). These observations would indicate that either export-promoting elements are quite plentiful, or that in the absence of any *cis*-elements, the default pathway for any given RNA is to be exported to the cytoplasm.

Recently we identified a motif found in certain *ftz* reporter plasmids, which promotes nuclear retention (Lee et al. 2015). This element, which is identical to the consensus 5′ splice site (5′SS) motif, was present in the multi-cloning region of the plasmid, just downstream from the *ftz* gene and upstream of the 3′ cleavage site, and was thus incorporated into the 3′ UTR of the mature *ftz* mRNA. When this motif was eliminated, the resulting intronless *ftz* mRNA was efficiently exported. These observations indicated that either the *ftz* RNA has an additional nuclear export-promoting element, or that when nuclear retention elements are eliminated, all mRNAs become substrates for nuclear export. These two possibilities were supported by the finding that UAP56, a central component of the TREX complex, is efficiently loaded onto *ftz* without the requirement of splicing (Taniguchi and Ohno 2008; Akef et al. 2013; Lee et al. 2015).

Here we analyze the export requirements of *ftz* and β*G* mRNAs. Our new results are consistent with the idea that the 3′ end of the β*G* gene inhibits nuclear export. According to our data, the activity of this nuclear retention element can be overridden by extending the RNA at the 5′ end or by splicing. Importantly, our new findings are consistent with the idea that in the absence of any *cis*-element an mRNA is exported to the cytoplasm, contrary to most currently accepted models of mRNA nuclear export.

## RESULTS

### Intronless *β-globin* mRNA contains a nuclear retention element

Previously we discovered that the intronless *fushi tarazu* (*c-ftz*-Δ*i*) mini-gene construct had a *cis*-element in its 3′ UTR region that inhibited nuclear export (Lee et al. 2015). This element actually mapped to a portion of the plasmid just downstream from *ftz* and was incorporated into the 3′ end of the mature mRNA. When this element was eliminated, the resulting RNA was efficiently exported to the cytoplasm despite the fact that it lacked an intron and was not spliced. We have also previously documented that certain elements that are found in the open-reading frame of some genes can also promote the nuclear export of *ftz* (Palazzo et al. 2007; Cenik et al. 2011). One such region is the signal sequence coding region (SSCR), which encodes a hydrophobic polypeptide that directs the newly synthesized protein to the secretory pathway. These sequences have particular features, such as a depletion of adenines, high GC-content, and an enrichment in certain motifs (Palazzo et al. 2007, 2013; Cenik et al. 2011). Recently we have determined that although SSCRs are required to promote the nuclear export of RNAs that are synthesized in vitro and directly microinjected into the nuclei of tissue culture cells, these elements do not affect the export of mRNAs that are transcribed in vivo from microinjected or transfected plasmids (Lee et al. 2015). For example, microinjected intronless *ftz* mRNA that lacks a nuclear retention element requires an SSCR or splicing for export, despite the fact that the same RNA generated from microinjected or transfected plasmids is exported without the need for either an SSCR or splicing.

Likewise, we had previously investigated whether the SSCR or *ftz* could promote the export of an intronless version of the *β-globin* gene (β*G-*Δ*i*) transcribed form microinjected plasmids. Surprisingly, we found mRNA produced from a gene containing an SSCR derived from the mouse MHC gene *h2kb* fused to β*G-*Δ*i* (*MHC-*β*G-*Δ*i*) was not efficiently exported (Akef et al. 2013). In contrast, mRNA produced from a fusion gene containing this SSCR, the *ftz* ORF (thus lacking its nuclear retention element), and β*G-*Δ*i* (*MHC-ftz-Δi-*β*G-*Δ*i*) was efficiently exported (Akef et al. 2013). In light of these results, we reasoned that *ftz* might contain a *cis*-element that promotes nuclear export.

**Figure 1.**
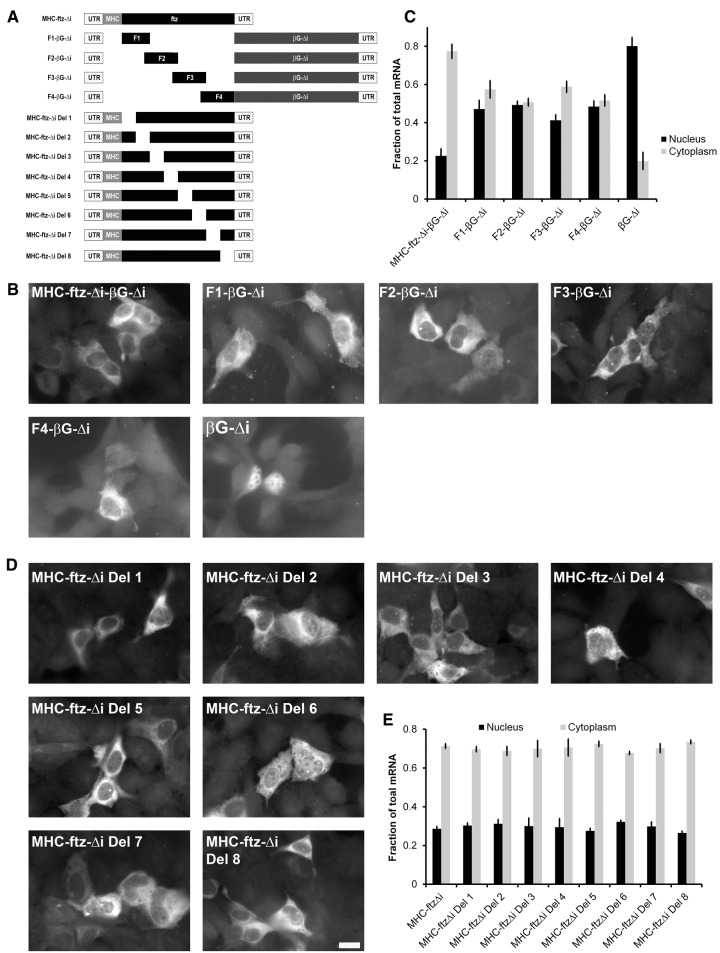
Fusions of *ftz* fragments to full-length βG mRNA are cytoplasmic at steady state. (*A*) A schematic representation of the different constructs that were used in this figure. (*B*–*E*) Plasmids containing the indicated constructs were transfected into human U2OS cells. After 14–18 h, the cells were fixed, permeabilized, and stained for mRNA using a FISH probe directed against β*G* (*B*,*C*) or the *MHC* SSCR (*D*,*E*). The cells were imaged (*B*,*D*) and mRNA distribution in the cytoplasm and nucleus was quantified (*C*,*E*). Each bar represents the average and standard error of three independent experiments, with each experiment consisting of at least 30 cells. Scale bar = 20 µm.

To investigate this possibility, we divided the *ftz* gene into four fragments and fused each of these to the 5′ end of β*G-*Δ*i* (fusion constructs, *F1-*β*G-*Δ*i* through *F4-*β*G-*Δ*i*, are shown in Figure 1A, for more details see Supplemental Table 1). The plasmids were transfected into U2OS cells and the mRNA was visualized by fluorescent in situ hybridization (FISH). To our surprise, all four of the constructs produced mRNA that was partially cytoplasmic (Fig. 1B,C). In contrast, β*G-*Δ*i* was mainly nuclear, and the fusion of full-length *MHC-ftz-*Δ*i* to β*G-*Δ*i* was primarily found in the cytoplasm (Fig. 1B,C), as we had previously seen (Akef et al. 2013).

Our results here could be explained by at least three models:

The simplest model is that each of the four *ftz* fragments contains a separate nuclear export-promoting element that work in an additive manner with each other or with the SSCR. However, when we investigated the distribution of mRNAs generated from various deletion *MHC-ftz-*Δ*i* deletion constructs (Fig. 1A), all of these were found in the cytoplasm at approximately the same level (Fig. 1D,E). Note that the previously identified nuclear retention element was not present in any of these constructs. Although this result does not rule out the idea that *ftz* has multiple export-promoting elements, this possibility became less likely.

A second model is that longer intronless mRNAs are simply more efficiently exported than shorter ones. This would explain the relative degrees of cytoplasmic accumulation where *MHC-ftz-*β*G-*Δ*i* is more cytoplasmic than any of the fusion constructs (*F1-*β*G-*Δ*i* through *F4-*β*G*-Δ*i*), which in turn are more cytoplasmic than *MHC-*β*G-*Δ*i* (Akef et al. 2013), which is slightly more cytoplasmic than β*G-*Δ*i*. Indeed, it had been previously found that certain RNA-binding proteins are differentially recruited to RNAs of different lengths and that these binding events can impact nuclear export (Masuyama et al. 2004; McCloskey et al. 2012). However, this second hypothesis would not be able to explain the efficient export of intronless *ftz*, which is approximately the same size as β*G-*Δ*i*.

**Figure 2.**
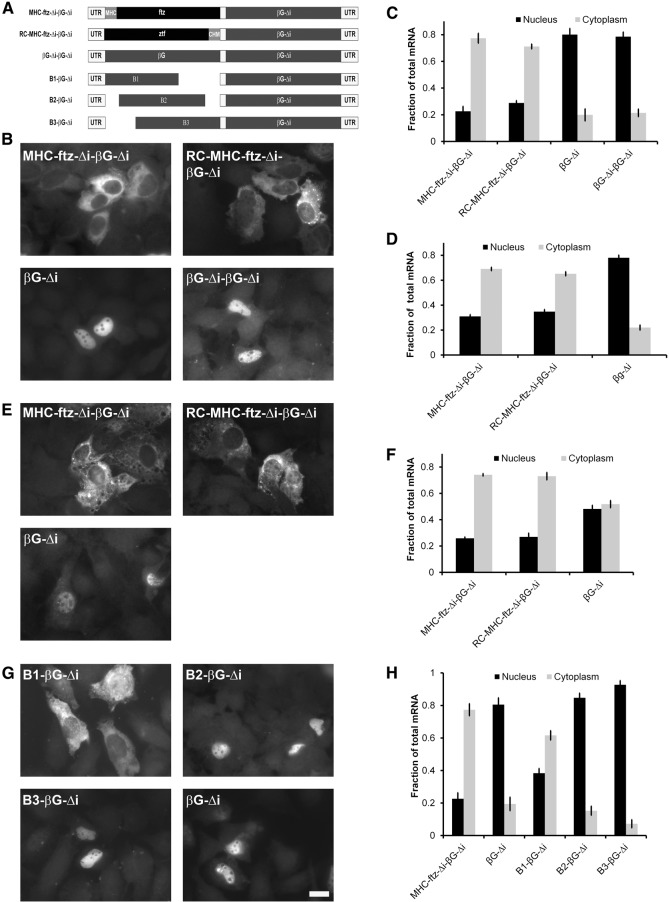
RNA insertions into the 5′ end of β*G* mRNA promotes nuclear export. (*A*) A schematic representation of the different fusion constructs that were used in this figure. (*B*,*C*) Plasmids containing the indicated constructs were transfected into human U2OS cells. After 14–18 h, the cells were fixed, permeabilized, and stained for mRNA using a FISH probe directed against β*G*. The cells were imaged (*B*) and mRNA distribution was quantified (*C*). Each bar represents the average and standard error of three independent experiments, each consisting of at least 30 cells. (*D*–*F*) Plasmids containing the indicated constructs were microinjected into the nuclei of U2OS (*D*) or NIH 3T3 (*E*,*F*) cells. After 20 min, cells were treated with α-amanitin and the newly synthesized mRNA was allowed to be exported for an additional 2 h. Cells were fixed, permeabilized, stained for mRNA using a FISH probe directed against β*G* and imaged. Examples of mRNA FISH staining in NIH3T3 cells are shown in *E*. The mRNA distribution was quantified for injected U2OS (*D*) and NIH3T3 (*F*), with each bar representing the average and standard error of three independent experiments, with each experiment consisting of at least 30 cells. (*G*,*H*) Plasmids containing the indicated constructs were transfected into human U2OS cells. After 14–18 h, the cells were fixed and permeabilized, stained for mRNA using a FISH probe directed against β*G*, imaged (*G*) and mRNA distribution was quantified (*H*). Each bar represents the average and standard error of three independent experiments, each consisting of at least 30 cells. Scale bar = 20 µm.

Finally, we considered the possibility that β*G* had a *cis*-element that retained the RNA in the nucleus, but that this activity could be overcome by either splicing (as is the case of the intron containing β*G*, [β*G-i*]) or by the incorporation of extra sequence into the 5′ end of the transcript. If this idea was correct, we reasoned that the incorporation of any random sequence into β*G-*Δ*i*, provided that this additional RNA did not contain any additional nuclear retention elements, should promote its export. Indeed we found that mRNA produced from the fusion of the reverse complement of *MHC-ftz-*Δ*i* to the 5′ end of β*G-*Δ*i* (to form *RC-MHC-ftz-*Δ*i-*β*G-*Δ*i*, see Fig. 2A; Supplemental Table 1) was distributed primarily to the cytoplasm in U2OS cells that were either transfected with a plasmid that contains this fusion gene (Fig. 2B,C) or that received this plasmid by microinjection (Fig. 2D). We obtained the same result in microinjected NIH 3T3 cells (Fig. 2E,F), indicating that this effect was not cell-type specific. Furthermore, this distribution was nearly identical to that of *MHC-ftz-Δi-*β*G-*Δ*i* (Fig. 2B–F). In contrast, a tandem repeat of β*G-*Δ*i* (β*G-Δi-*β*G-*Δ*i*, see Fig. 2A; Supplemental Table 1) was retained in the nucleus, to the identical extent as the original β*G-*Δ*i* (Fig. 2B,C). This result can be explained by the fact that this longer mRNA (β*G-Δi-*β*G-*Δ*i*) now had a second putative nuclear retention element.

**Figure 3.**
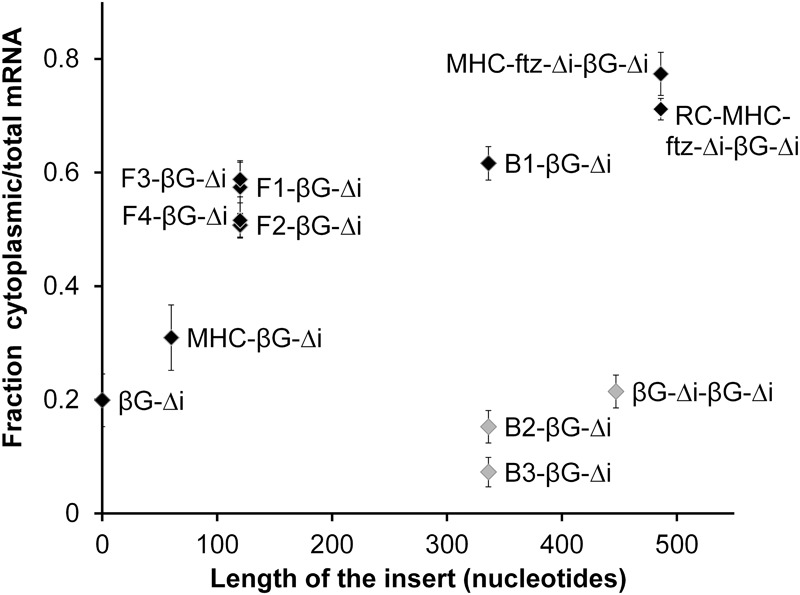
Extending the length of β*G-*Δ*i* mRNA promotes its nuclear export. For each of the fusion constructs in Figures 1A, 2A, and 4A, the length of insertion added upstream of the β*G-*Δ*i* ORF mRNA is plotted against the percentage of cytoplasmic to total mRNA. Consistent with the idea that the 3′ terminal portion of the β*G-*Δ*i* mRNA contains a nuclear retention element, constructs that contain one copy of this region are in black and generally follow one trend, while constructs that contain two copies of this region are in gray, are much more nuclear and follow a different trend. Note that the data were obtained from different experiments that were not all done in parallel but all performed in U2OS cells.

With the data that we collected thus far, we could not rule out the possibility that export-promoting elements were found throughout *ftz-*Δ*i* and in the reverse complement of *MHC-ftz-Δi.* We reasoned that of all the transcripts that we have tested thus far, the most unlikely place to find an export-promoting element would be in β*G-*Δ*i* itself. We thus started off with the tandem construct (β*G-*Δ*i*-β*G-*Δ*i*) and deleted portions of the first copy of β*G-*Δ*i* with the goal of eliminating its nuclear retention element (see *B1-*β*G-*Δ*i* through *B3-*β*G-*Δ*i* Fig. 2A; Supplemental Table 1). Since it appeared that the size of the insert dictated the level of export, we made only small deletions to the first copy of β*G-*Δ*i*. We found that one of the three new fusion constructs (*B1-*β*G-*Δ*i*) was indeed exported (Fig. 2G,H). When the export versus the total RNA length of the various β*G-*Δ*i* fusion constructs was plotted, we saw a correlation between these two variables (Fig. 3, see black data points). The only exceptions were the β*G-Δi-*β*G-*Δ*i* tandem construct, *B2-*β*G-*Δ*i* and *B3-*β*G-*Δ*i* (Fig. 3, see gray data points), likely because these all had two copies of the nuclear retention element.

From these experiments we concluded that β*G-*Δ*i* has a nuclear retention element near its 3′ end. Our data suggested that the activity of this element could be inhibited by simply extending the length of the RNA at the 5′ end, or by incorporating an intron in the pre-mRNA. While it is still possible that the B1 region of β*G-*Δ*i* has a nuclear export element, a counter-acting *cis*-element would need to be invoked to explain why β*G-*Δ*i* is not exported in the first place.

### Mapping the nuclear retention elements in β*G*-Δ*i*

**Figure 4.**
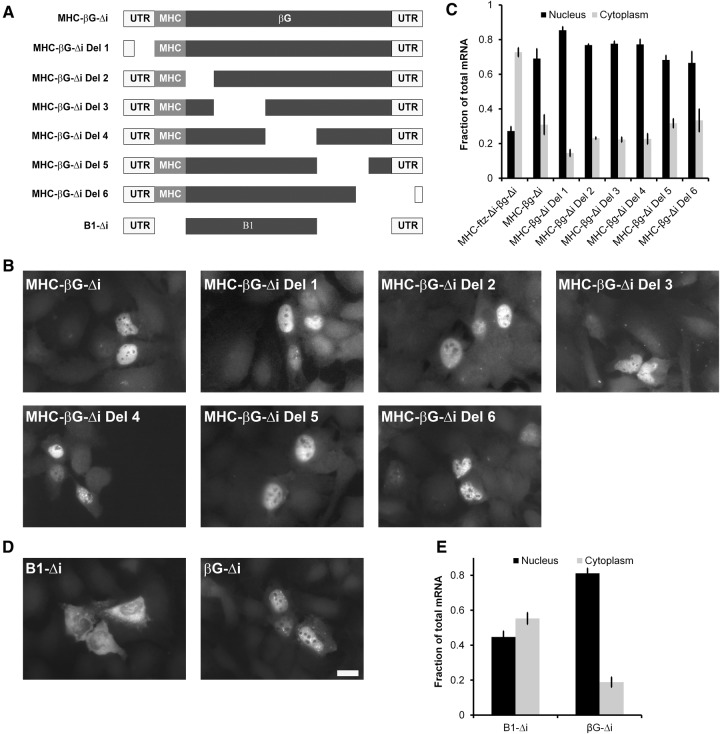
The 3′ end of β*G-*Δ*i* is required for nuclear retention. (*A*) A schematic representation of the different constructs that were used in this figure. (*B–E*) Plasmids containing the indicated constructs were transfected into human U2OS cells. After 14–18 h, the cells were fixed, permeabilized, and stained for mRNA using a FISH probe directed against the MHC SSCR (*B*,*C*) or β*G* (*D*,*E*). The cells were imaged (*B*,*D*) and mRNA distribution was quantified (*C*,*E*). Each bar represents the average and standard error of three independent experiments, each experiment consisting of at least 30 cells. Scale bar = 20 µm.

To better define the retention element, we tested various deletion constructs of β*G-*Δ*i* (a schematic illustration of the constructs is shown in Fig. 4A, also see Supplemental Table 1). Since all of the constructs contained the MHC SSCR, we could use FISH probes against this common region to visualize all of the deletion constructs. We found that all of these constructs (*MHC-*β*G-Δi Del1* through *Del6*) were distributed primarily to the nucleus (Fig. 4B,C). One possible explanation for this result is that there are two redundant nuclear retention elements, both of which map to the region that was deleted to form the *B1-*β*G-*Δ*i* construct, and thus residing near the 3′ end.

To confirm that this is the case we created a new construct, *B1-*Δ*i* construct (Fig. 4A). The *B1* region was identical to the first part of the *B1-*β*G-*Δ*i* tandem construct (see Fig. 2A; Supplemental Table 1) and was missing a 215-nucleotide (nt) fragment that matched the portions of β*G* ORF that were deleted in *MHC-*β*G-Δi Del5* and *Del6* (Fig. 4A; Supplemental Table 1). Since our β*G* FISH probe could detect this construct, we did not need to add the MHC SSCR to the 5′ end. Confirming our suspicions, we found that *B1-*Δ*i* mRNA was more efficiently exported than β*G-*Δ*i* (Fig. 4D,E). Interestingly, the *B1-*Δ*i* mRNA was still not as efficiently exported as *ftz-*Δ*i* or the *MHC-ftz-Δi-*β*G-*Δ*i* fusion. This leaves open the possibility that *ftz-*Δ*i* may indeed contain nuclear export-promoting elements. Alternatively, the *B1* region may contain additional *cis*-elements that alter mRNA export dynamics.

### The activity of the β*G* nuclear retention element(s) can be inhibited by splicing

**Figure 5.**
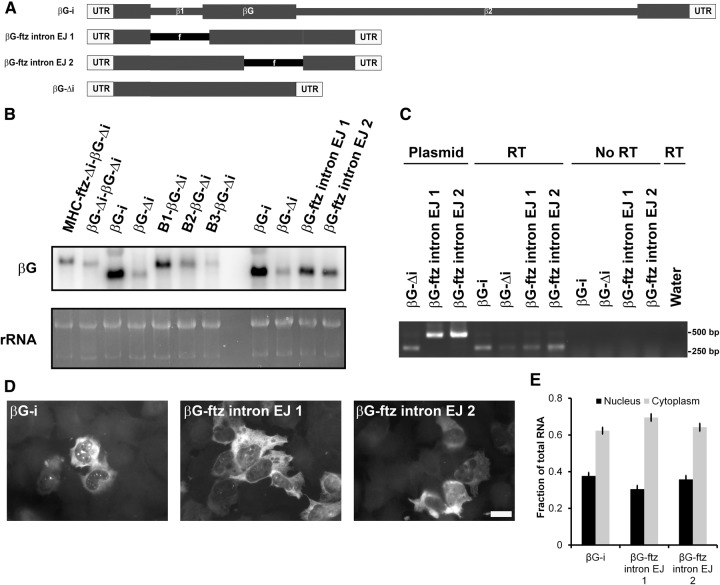
Splicing is sufficient to overcome the nuclear retention element present in β*G-*Δ*i* mRNA. (*A*) A schematic representation of the intron containing constructs used in this figure. (*B*–*E*) Plasmids containing the indicated constructs were transfected into human U2OS cells. After 14–18 h, RNA was isolated (*B*,*C*) or cells were fixed, permeabilized, and stained for mRNA using a FISH probe directed against β*G* (*D*,*E*). (*B*) Northern blot using β*G*-specific probes. Total RNA (mostly comprising of rRNA) was stained with ethidium bromide as a loading control. (*C*) Splicing efficiency of the different reporters was assessed by RT–PCR using RNA isolated from transfected cells and primers that anneal to the first and third exons of β*G-*Δ*i* mRNA (see “RT” reactions). To determine the size of putative unspliced RNAs, PCR reactions were performed on purified plasmid DNA (see “Plasmid”). To control for the amplification of transfected plasmid DNA, amplification of isolated cellular RNA was performed without a reverse transcription step (“No RT”). (*D*) Representative example of imaged cells. Scale bar = 20 µm. (*E*) mRNA distribution was quantified with each bar representing the average and standard error of three independent experiments, and each experiment consisting of at least 30 cells.

Having uncovered evidence of a nuclear retention element localized to the 3′ end of the β*G-*Δ*i* mRNA, we wanted to reinvestigate how splicing suppresses this activity. The human β*G* pre-mRNA has two introns (Fig. 5A), the second one residing within the 215-nt sequence that was required for nuclear retention. We reasoned that either the nuclear retention activity could be overcome by the splicing of any intron, that the endogenous β*G* introns have some specialized activity that counteracts this retention element, or that the second intron simply disrupted the retention element in the pre-mRNA. To distinguish between these three possibilities, we inserted the *ftz* intron into β*G-*Δ*i* so that it occupies a position where either the first or second endogenous introns normally reside (these being the first or second exon junctions—EJ1 or EJ2, see β*G-ftz intron EJ1* and *EJ2*, Fig. 5A). Constructs containing the *ftz* intron at either position were efficiently spliced as assayed by Northern blot (Fig. 5B) and RT-PCR (Fig. 5C). Furthermore, both mRNAs were efficiently exported (Fig. 5D,E) comparable to a version of spliced β*G* mRNA (β*G-i)* that was generated from a pre-mRNA containing its two endogenous introns (β*G-i*).

From these results we conclude that there is likely nothing special in the endogenous β*G* introns that allow them to overcome the nuclear retention element. Our data also indicate that the introns do not promote export by disrupting the nuclear retention element in the pre-mRNA, as this element is intact in the β*G-ftz intron EJ1* construct. Rather, our results suggest that that splicing per se, regardless of where the splicing occurs, is sufficient to overcome the activity of the nuclear retention element.

### Nuclear/cytoplasmic distribution correlates with mRNA levels

**Figure 6.**
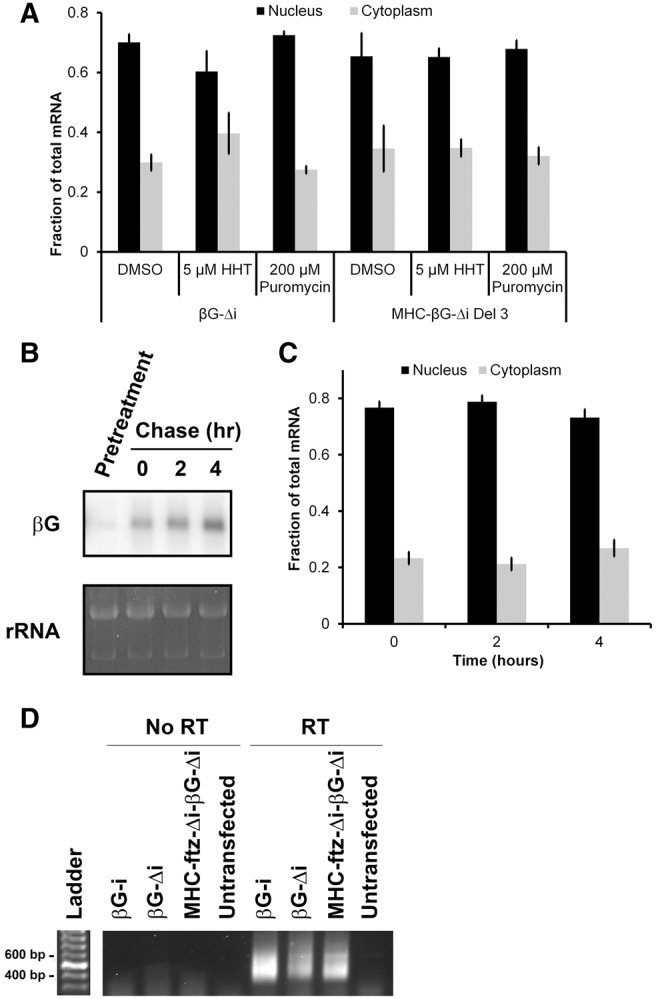
The majority of the β*G-*Δ*i* mRNA that is present at steady state is stable and retained in the nucleus. (*A*) To test whether β*G-*Δ*i* is a substrate for NMD, U2OS cells were transfected for 6 h, then treated with either DMSO, puromycin, or HHT at levels that completely inhibit translation (see Cui et al. 2012) for a further 8 h. Cells were then fixed, permeabilized, stained for mRNA using a FISH probe directed against β*G* for β*G-*Δ*i* mRNA, or *MHC* SSCR for *MHC-*β*G-*Δ*i Del 3*, and the levels of nuclear and cytoplasmic RNA were quantified (*B*,*C*). U2OS cells were transfected for 14 h, then treated with α-amanitin for the indicated times. Then, either RNA was collected and analyzed by Northern blot using β*G*-specific probes (*B*) or cells were fixed, permeabilized, stained for mRNA using a FISH probe directed against β*G* and the levels of nuclear and cytoplasmic RNA were quantified (*C*). To ensure that the α-amanitin inhibited transcription, cells were first treated with drug, then transfected (“Pretreatment”). Total RNA (mostly comprising of rRNA) was stained with ethidium bromide as a loading control. Each bar in *A* and *C* represents the average and standard error of three independent experiments, each of which consists of at least 30 cells. (*D*) Cells were transfected with the indicated constructs and RNA was isolated after 14 h. To ensure that the specificity of the detected signal, RNA was also isolated from untransfected cells. The length of their poly(A) tails was assessed using ePAT as described previously (Jänicke et al. 2012). Reverse transcription reactions were performed with either the reverse transcriptase added, “RT”, or not added to the reactions, “No RT.”

While examining our FISH images, we noticed that all the versions of β*G* mRNA that were primarily localized to the nucleus also gave week fluorescent signals. To further study this we monitored the levels of each mRNA by Northern blot (Fig. 5B). Indeed any β*G* mRNA that was poorly exported (β*G-*Δ*i*, β*G-Δi-*β*G-*Δ*i*, *B2-*β*G-*Δ*i*, and *B3-*β*G-*Δ*i*) also was present at low levels in comparison to the well-exported mRNAs. Thus although we have been describing the activity present at the 3′ end of the β*-G* transcript as promoting nuclear retention—based partially on the reports of other groups that have investigated this transcript (Valencia et al. 2008; Lei et al. 2011, 2012)—it is entirely possible that these transcripts are exported to the cytoplasm and then rapidly degraded, resulting in a low cytoplasmic/nuclear ratio. This opened the possibility that the transcripts are being degraded by nonsense-mediated decay (NMD), which eliminates mRNAs that have premature stop codons and/or aberrant 3′ UTRs (Schweingruber et al. 2013). NMD is activated by translating ribosomes and is inhibited by extended treatments with translation inhibitors. However, treatment of cells with two different translation inhibitors, puromycin and homoharingtonine (HHT) at concentrations that effectively inhibit translation in U2OS cells (Cui et al. 2012), had no effect on the cytoplasmic distribution of either β*G-*Δ*i* or *MHC-*β*G-Δi Del3*, which has a premature termination codon (Fig. 6A).

Thus we conclude that the transcripts are not substrates for NMD.

### The majority of β*G-*Δ*i* at steady state is stable and retained in the nucleus

It remained possible that the high nuclear/cytoplasmic ratio of β*G-*Δ*i* was due to the fact that this mRNA was exported and rapidly degraded in the cytoplasm. For this to be true, mRNA decay would have to be at least as fast as nuclear export. The half rate of export (the time it takes to export half of all the nuclear mRNA) for spliced β*G* is ∼1 h (Valencia et al. 2008; A Akef and AF Palazzo, unpubl.). To determine whether the distribution of β*G-*Δ*i* was due to a high turnover rate, we measured its half-life by treating transfected cells with the transcriptional inhibitor α-amanitin at levels which completely block transcription of microinjected plasmids (Gueroussov et al. 2010), and monitored the amount of β*G-*Δ*i* mRNA remaining after various time points. Note that α-amanitin treatment completely blocks transcription within the first 5 min of treatment (Gueroussov et al. 2010; A Akef and A Palazzo, unpubl.). Surprisingly, we could not detect any turnover of the mRNA even after a 4 h treatment (Fig. 6B), despite the fact that a pretreatment with α-amanitin prevented the synthesis of this transcript (“Pretreatment,” Fig. 6B). Indeed it had been previously reported that the half-life of β*G-*Δ*i* ranges from 8 to 15 h (Lu and Cullen 2003). We found that this mRNA remained in the nucleus throughout the entirety of the time course and was not exported (Fig. 6C). Thus most of the β*G-*Δ*i* mRNA that is present in the cell at steady state is relatively stable, yet fails to be exported to the cytoplasm. We obtained similar results when we investigated how the 5′ SS motif affected the nuclear export of *ftz* mRNA (Lee et al. 2015). This motif promoted the rapid turnover of about half of the mRNA in the first hour, while the portion that evaded this initial decay step built up over time in the nucleus and represented the majority of the *ftz* mRNA that was present at steady state. Like β*G-*Δ*i*, this stable nuclear fraction of *ftz* mRNA remained trapped in the nucleus and was not a substrate for export.

From these results we conclude that the element present at the 3′ end of the β*G-*Δ*i* mRNA acts as a nuclear retention element. It is also likely that this element promotes the rapid decay of a fraction of the newly synthesized mRNA, as we had previously observed with the 5′ SS motif (Lee et al. 2015).

### The length of the poly(A)-tail is unaltered by the nucleocytoplasmic distribution of the mRNA

Previous studies have suggested a correlation between splicing and the 3′ processing of β*G* RNA (Millevoi et al. 2002; Lu and Cullen 2003). Therefore, we wanted to investigate whether the lack of export was due to alterations in the poly(A)-tail length. Using the extension poly(A) test (ePAT) method, we compared the 3′ processing of β*G-*Δ*i*, an mRNA that is neither spliced nor exported, β*G-i*, an mRNA that is both spliced and exported, and *MHC-ftz-Δi-*β*G-*Δ*i*, which is not spliced but exported to the cytoplasm (see Fig. 2A). As shown in Figure 6D, the poly(A) tails of β*G-i*, β*G-Δi,* and *MHC-ftz-Δi-*β*G-*Δ*i* mRNAs were of similar size (∼120–150 nts, see Materials and Methods section). No signal was obtained from untransfected cells or when the reverse transcriptase step was omitted.

From these results, we conclude that the poly(A)-tail length did not significantly change between spliced and unspliced β*G* mRNAs. Importantly, these results rule out the possibility that the nuclear retention of β*G-*Δ*i* can be simply attributed to inefficient polyadenylation.

### UAP56 is recruited to the β*G-*Δ*i* mRNA

**Figure 7.**
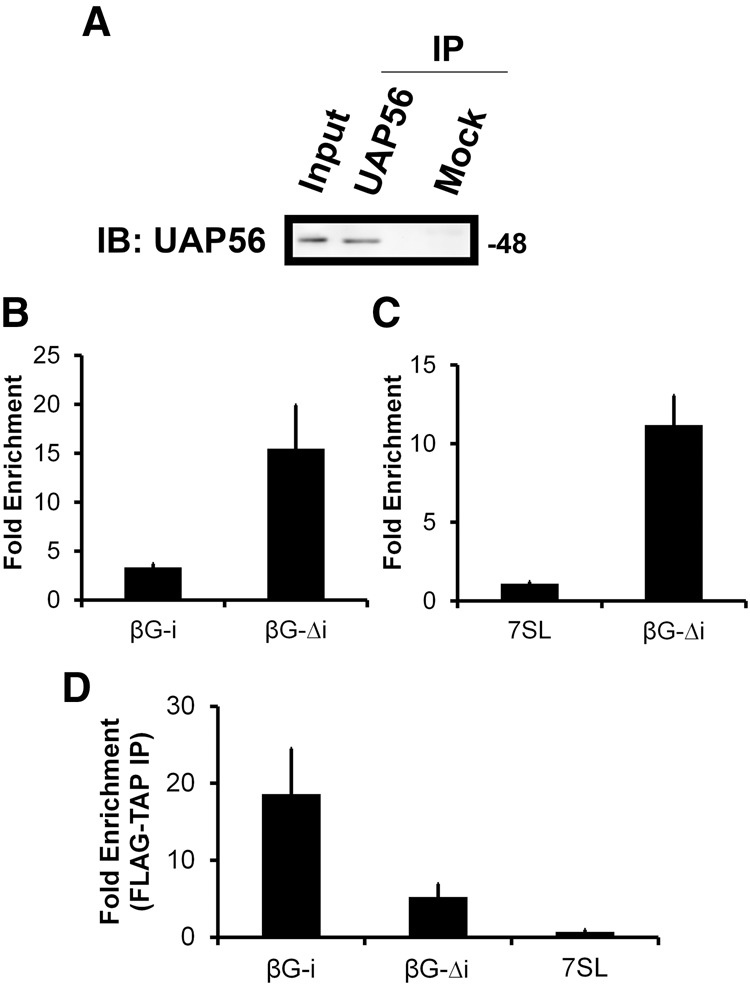
Strong UAP56-association and weak TAP-association with β*G-*Δ*i* mRNA in vivo. (*A*) UAP56 was immunoprecipitated from U2OS lysates using rat anti-UAP56 antibodies prebound to protein G sepharose. The immunoprecipitates were analyzed by immunoblotting for UAP56. Rat preimmune serum was used in the mock immunoprecipitation reaction. (*B*,*C*) U2OS cells were transfected with plasmids containing β*G-*Δ*i* (*B*,*C*) or β*G-i* (*B*). After 14–18 h, cell lysates were collected and immunoprecipitated with rat anti-UAP56 antibodies or rat preimmune serum. RNA was collected from fractions and converted to cDNA using β*G*-specific primers (*B*) or random hexamers (*C*). The fold enrichment of mRNAs in anti-UAP56 over preimmune precipitates was quantified by RT-qPCR. Each bar represents the average and standard error of three independent experiments. (*D*) U2OS cells were cotransfected with plasmids containing FLAG-TAP and either β*G-*Δ*i* or β*G-i*. After 14–18 h, cell lysates were collected and immunoprecipitated with beads conjugated to anti-FLAG antibodies or protein A. RNA was collected from fractions and converted to cDNA using random hexamers. The fold enrichment of mRNAs in the FLAG precipitates over protein A precipitates was quantified by RT-qPCR. Each bar represents the average and standard deviation of two independent experiments.

To gain further insight into how this nuclear retention element functions, we decided to reevaluate whether components of the TREX complex are recruited to the RNA in a splicing-dependent manner. As we have done previously (Akef et al. 2013; Lee et al. 2015), we expressed various reporters in U2OS cells and then from these lysates immunoprecipitated UAP56 (see Fig. 7A), a central component of the TREX complex, and analyzed the precipitated RNA by RT-qPCR. To our surprise, we found that β*G-*Δ*i* was more highly enriched in the UAP56 precipitates than the spliced version of the transcript (β*G-i*) (Fig. 7B). This coprecipitation was likely specific because 7SL, a highly abundant ncRNA which is exported by exportin-5 in vertebrates independently of the TREX complex (Takeiwa et al. 2015), was not significantly enriched in the precipitates (Fig. 7C). This result suggests that the retention element traps the mRNA in a UAP56-bound state.

Next we examined the recruitment of TAP (also known as NXF1) to β*G-*Δ*i*. Due to the fact that we do not have an antibody that reliably immunoprecipitates endogenous TAP, we coexpressed a FLAG-tagged version of the construct along with β*G-*Δ*i* or β*G-i* in U2OS cells and precipitated this exogenous protein from the lysates using anti-FLAG antibodies, as we had done previously (Lee et al. 2015). We found that although β*G-*Δ*i* mRNA was present in the immunoprecipitates, it was there at much lower levels than a spliced form of β*G* (Fig. 7D). This result may suggest that β*G*Δ*i* mRNA is not trapped in TAP-associated form, as what we saw for UAP56. As expected, 7SL was not significantly enriched in the TAP precipitates.

In summary, these results suggest that the presence of introns, and hence their splicing, is not the sole determinant of UAP56 recruitment to the β*G* mRNA, as the standard model would suggest. Our data suggest that the retention element in β*G-*Δ*i* may prevent efficient TAP recruitment, and perhaps this recruitment event becomes more efficient by the act of splicing. However, at this point we cannot rule out the possibility that this retention element recruits some dominant-acting complex that inhibits export regardless of whether or not TAP is recruited to the RNA.

## DISCUSSION

**Figure 8.**
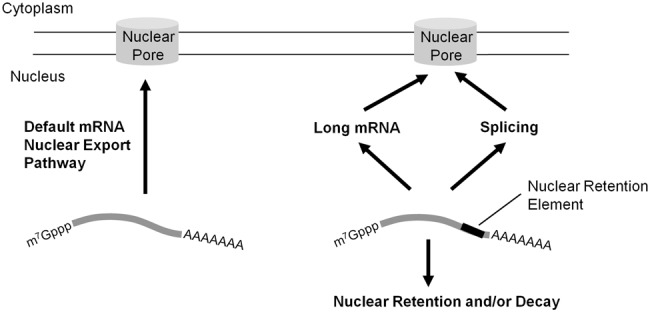
Model of how the β*G* nuclear retention element affects mRNA nuclear export.

There has been an ongoing debate as to whether splicing is required for the efficient export of mRNAs in vertebrate cells. Underlying this debate is the question of whether mRNAs are natural substrates for nuclear export by the TREX complex in the absence of either splicing, or any other export-promoting element. The idea that nuclear export requires specialized signals is based on two pieces of data. First, the export of certain model mRNAs, such as *ftz* and *β-globin*, was found to be greatly enhanced by splicing (Luo and Reed 1999; Valencia et al. 2008). Second, the recruitment of nuclear export factors to the mRNA in HeLa-derived nuclear extracts was also found to be enhanced by splicing (Zhou et al. 2000; Masuda et al. 2005; Cheng et al. 2006; Dufu et al. 2010; Chi et al. 2013). There are, however, a few problems with this view. First, many cDNA-derived mRNAs are well exported despite the fact that their endogenous genes contain introns (Palazzo and Akef 2012). Second, TREX components are known to associate with intronless mRNAs in vivo and are required for their export (Rodrigues et al. 2001; Taniguchi and Ohno 2008; Hautbergue et al. 2009; Akef et al. 2013; Lee et al. 2015). Our new study, coupled with a previous report (Lee et al. 2015) now resolves some of this debate. It appears that certain cDNA-derived mRNAs, including many of the ones being studied in various nuclear export labs, contain nuclear retention elements. Although we cannot rule out the possibility that a wide range of nuclear-export promoting elements exist in addition to these retention elements, on the whole we now believe that in the absence of any specialized *cis*-element, mRNAs are substrates for nuclear export (Fig. 8). This also resolves an apparent difference that emerged between mRNA export in vertebrates and yeast, where splicing is known to be dispensable for nuclear export.

In light of our new understanding, we can now reinterpret some other published data. It had been previously reported that the fusion of 16 CAR-E motifs to the 5′ end of the β*G-*Δ*i* mRNA promoted export, while the fusion mutant versions of these CAR-Es did not (Lei et al. 2012). Our new data suggest that a simple extension of the β*G-*Δ*i* mRNA promotes export by overcoming nuclear retention elements present at the 3′ end of the transcript (Fig. 8). Looking closely at the mutant CAR-E insertions, they contained a high level of pyrimidines, and were found to be associated with poly-pyrimidine track-binding (PTB) protein as assessed by mass spectrometry analysis (Lei et al. 2012). Interestingly, it has been previously reported that PTB inhibits nuclear RNA export (Roy et al. 2013). Thus it is possible that the mutant CAR-Es acted as nuclear retention elements.

It is likely that these nuclear retention elements are widely distributed, as many cDNA-derived RNAs are known to be retained in the nucleus (some examples include the major late adenovirus RNA [Luo et al. 2001], a fragment of the Xenopus Smad 3 gene [Valencia et al. 2008], Slu7 and DDX3 [Lei et al. 2012]). There have also been quite a few reports describing how the association of certain RNA-binding proteins to an RNA promotes its nuclear retention. Factors that are known to have this activity include not only PTB, but also hnRNP A2 (Lévesque et al. 2006), hnRNP U (Hacisuleyman et al. 2014), U2AF65, and U170K (Takemura et al. 2011). Our data suggest that although splicing may not be required for the export of every RNA, splicing does help to export those that have certain nuclear retention elements, such as the one found in the β*G* gene (Fig. 7). Interestingly, splicing does not effectively promote the export of mRNAs that contain a 5′ splice site motif in their 3′ UTR (Lee et al. 2015), or nuclear retention elements found in certain lncRNAs (Hacisuleyman et al. 2014).

Large-scale analyses of mRNA distribution have indicated that a significant fraction of human mRNAs is predominantly distributed to the nucleus at steady state (Djebali et al. 2012). This diverse distribution of mRNA has a profound effect on the proteome of human cells. Ultimately a deeper understanding of all the *cis-*elements that govern nuclear retention and export will help us to understand how various mRNAs are either exported or retained in the nucleus.

## MATERIALS AND METHODS

### Plasmid constructs

*MHC-ftz-*Δ*i*, c-*ftz-Δi, c-ftz-i, MHC-*β*G-Δi, MHC-ftz-Δi-*βG-Δ*i*, β*G-*Δ*i*, β*G-i*, in pCDNA3 were described previously (Palazzo et al. 2007; Valencia et al. 2008; Akef et al. 2013; Lee et al. 2015). The series of *MHC-ftz*Δ*i* and *MHC-*β*G-*Δ*i* deletions were performed using primers that anneal upstream and downstream of the sequence to be deleted. After the PCR, the products were treated with DpnI, polynucleotide kinase (PNK) and ligated using T4 DNA ligase. The *F1-*β*G-*Δ*i* to *F4-*β*G-*Δ*i* constructs were generated using restriction-free cloning (van den Ent and Löwe 2006), where the respective sequence was amplified using *c-ftz-*Δ*i* as a template. The PCR products were run on a gel and purified using DNA extraction kit (Qiagen). The purified products were inserted right after the start codon of β*G-*Δ*i* using a PCR reaction. The *RC-MHC-ftz-Δi-*β*G-*Δ*i* and β*G-*Δ*i*-β*G-*Δ*i* were constructed by amplifying *MHC-ftz-*Δ*i* or β*G-*Δ*i* using a reverse primer that contained a HindIII site just upstream of the stop codon. The PCR products were digested with HindIII and ligated into β*G-*Δ*i* pCDNA3 that was cut with the same enzyme. The series of *B1-*β*G-*Δ*i* to *B3-*β*G-*Δ*i* constructs were generated by amplifying 330-nt sequences from β*G-*Δ*i* using a reverse primer that contained a KpnI site and ligated into β*G-*Δ*i* pcDNA 3 that was cut with the same enzyme. To generate *B1-*Δ*i* construct, a forward primer that anneals downstream from the XhoI site and a reverse primer that anneals to the end of the B1 insert in the *B1-*β*G-*Δ*i* construct were used in a PCR reaction that had β*G-*Δ*i* as a template. The PCR products were DpnI treated, PNK treated, and ligated using T4 DNA ligase. The β*G-ftz intron EJ1* and β*G-ftz intron EJ2* constructs were generated by amplifying the intron of *ftz-i* using primers that anneal at their 5′ ends to the endogenous exon–exon junctions 1 and 2 of β*G-*Δ*i*. These introns were then inserted into β*G-*Δ*i* by restriction-free cloning PCR amplification. DH5α *E. coli* cells were transformed with the cloned plasmids. A description of the various constructs used in the study is shown in Supplemental Table 1.

### Cell lines and tissue culture

Human osteosarcoma (U2OS) cells were maintained in high glucose DMEM (Wisent) containing 10% FBS (Wisent) and antibiotics (Sigma). Mouse fibroblasts (NIH 3T3) were maintained in high glucose DMEM containing 10% calf serum (Wisent) and antibiotics (Sigma). The following drugs were used: puromuycin (Sigma) was used at 200 µm, homoharringtonine (Tocris Bioscience) was used at 5 µm, and α-amanitin (Sigma) was used at 1 µg/mL.

### Transfection, microinjection, FISH, and immunostaining

Cells were plated on 22 × 22 mm acid-washed cover slips (VWR) in 35 mm mammalian tissue culture dishes (Thermo Scientific) for 24 h prior to injection or transfection. Cells were transfected using GenJet in vitro DNA Transfection Reagent for U2OS (SignaGen Laboratories) according to the manufacturer's protocol. DNA microinjections were performed as previously described (Gueroussov et al. 2010). Fluorescence in situ hybridization (FISH) mRNA staining was performed as previously described (Gueroussov et al. 2010). The probe oligonucleotide sequences used included anti-*ftz* (GTCGAGCCTGCCTTTGTCATCGTCGTCCTTGTAGTCACAACAGCCGGGACAACACCCCAT), anti*-*β*G* (CTTCATCCACGTTCACCTTGCCCCACAGGGCAGTAACGGCAGACTTCTCCTCAGGAGTCA), and anti-MHC probe (TCTGAGTCGGAGCCAGGGCGGCCGCCAACAGCAGGAGCAGCGTGCACGGT).

### Imaging and image analysis

Cells were imaged using a fluorescence microscope (Nikon) as previously described (Gueroussov et al. 2010). Image analysis, including the quantification of mRNA export, was performed using Nikon Imaging Software (NIS) Elements Advanced Research (Nikon) as previously described (Gueroussov et al. 2010).

### Northern blotting

Total RNA was isolated from transfected cells using Tri reagent (MRC) according to the manufacturer's protocol. The RNA was separated on a denaturing 1% agarose gel in MOPS buffer. The gel was stained with ethidium bromide to visualize total RNA (mostly comprising of rRNA) as a loading control. The RNA was transferred overnight to a nitrocellulose membrane. Subsequently, the membrane was UV crosslinked and the RNA hybridized to a ^32^P-labeled probe overnight in Church buffer 65°C. The membrane was imaged using a Typhoon phosphoimager system. The probe was generated first restricting the plasmid using HindIII and XhoI (NEB), gel purifying the restricted fragment and using Prime a gene kit (Promega) in the presence of α^32^P-dATP.

### Endpoint RT-PCR, ePAT

To assess the efficiency of splicing for intron containing mRNAs, total RNA was isolated from transfected cells. The RNA was reverse-transcribed using SuperscriptIII (Invitrogen) according to the manufacturer's protocol. Subsequently, the cDNA was amplified using primers that are upstream and downstream of the introns. The amplicons were separated on an agarose gel and stained with ethidium bromide. ePAT was performed as previously described (Jänicke et al. 2012). Briefly, the RNA was isolated using Tri Reagent (Molecular Research Center) and extended using Klenow (NEB). Subsequently, the RNA was reverse-transcribed using SuperscriptIII (Invitrogen). The cDNA was amplified and separated on an agarose gel that was stained with ethidium bromide. Poly(A)-tail length was estimated by subtracting the region of β*G* amplified upstream of the 3′ cleavage site as mapped by 3′ RACE (265 nts) from the estimated amplicon size. Note that mRNA isolated from cells that were transfected with plasmids containing β*G-i* or β*G-*Δ*i* had the same 3′ cleavage site as determined by 3′ RACE.

### RNA immunoprecipitation

RNA IP was performed as previously described (Akef et al. 2013; Lee et al. 2015). FLAG-TAP plasmid (inserted in p3xFlag-CMV-10 vector, as previously described [Lykke-Andersen et al. 2001]) was cotransfected with the various reporter plasmids. For UAP56 pulldowns, cell lysate from transfected cells was incubated for 10–14 h with either rat anti-UAP56 antibodies (Yamazaki et al. 2010) or rat preimmune serum prebound to protein G sepharose (Invitrogen). For FLAG-TAP pulldowns, cell lysate from transfected cells was incubated with ANTI-FLAG M2 Affinity Gel beads (A2220, Sigma-Aldrich) or protein A beads for 2.5 h. cDNA was synthesized using SuperScript III (Invitrogen) according to the manufacturer's protocol. qPCR was performed by mixing the cDNA with Power Sybr Green Master Mix (Invitrogen) and the reaction was run on a CFX384 Touch Real Time PCR Detection System (Bio-Rad). The efficiency of the IP reaction was confirmed by separating the cell lysate and immunoprecipitates by SDS–PAGE, transferring the proteins to nitrocellulose and immunoblotting for UAP56 using rabbit anti-UAP56 antibodies (Sigma).

## SUPPLEMENTAL MATERIAL

Supplemental material is available for this article.

## Supplementary Material

Supplemental Material
