# Supplementary material for: Splicing promotes the nuclear export of β-globin mRNA by overcoming nuclear retention elements
Source: RNA. 2015 Nov;21(11):1908–20. doi: 10.1261/rna.051987.115 (PMC4604431; doi:10.1261/rna.051987.115)
Supplement: Supplemental Material [file supp_21_11_1908__index.html]

Splicing promotes the nuclear export of β-globin mRNA by overcoming nuclear retention elements — Splicing promotes the nuclear export of β-globin mRNA by overcoming nuclear retention elements — Supplemental Material 

# Splicing promotes the nuclear export of *β-globin* mRNA by overcoming nuclear retention elements

## Supplemental Material

**Files in this Data Supplement:**

- Supp Table 1.docx
