# Supplementary material for: Splicing promotes the nuclear export of β-globin mRNA by overcoming nuclear retention elements
Source: RNA. 2015 Nov;21(11):1908–20. doi: 10.1261/rna.051987.115 (PMC4604431; doi:10.1261/rna.051987.115)
Supplement: Supplemental Material [file supp_051987.115_SuppTable1.docx]

**Table 1: A description of the constructs used in this study.**

| **Construct** | **Description** |
| --- | --- |
| MHC-ftz-∆i | See Palazzo et al. (2007) |
| c-ftz-∆i | See Palazzo et al. (2007) |
| MHC-βg-Δi | See Akef et al. (2013) |
| βG-∆i | See Valencia et al. (2008) |
| βG-i | See Akef et al. (2013) |
| c-ftz-i | See Palazzo et al. (2007) |
| F1-βG-∆i | Sequence -20 to 99 of c-ftz-∆i inserted downstream of nucleotide 3 of βG-∆i |
| F2-βG-∆i | Sequence 80 to 199 of c-ftz-∆i inserted downstream of nucleotide 3 of βG-∆i |
| F3-βG-∆i | Sequence 180 to 299 of c-ftz-∆i inserted downstream of nucleotide 3 of βG-∆i |
| F4-βG-∆i | Sequence 280 to 399 of c-ftz-∆i inserted downstream of nucleotide 3 of βG-∆i |
| MHC-ftz-∆i Del 1 | Sequence 67 to 116 deleted from MHC-ftz-∆i |
| MHC-ftz-∆i Del 2 | Sequence 117 to 166 deleted from MHC-ftz-∆i |
| MHC-ftz-∆i Del 3 | Sequence 167 to 216 deleted from MHC-ftz-∆i |
| MHC-ftz-∆i Del 4 | Sequence 217 to 266 deleted from MHC-ftz-∆i |
| MHC-ftz-∆i Del 5 | Sequence 267 to 316 deleted from MHC-ftz-∆i |
| MHC-ftz-∆i Del 6 | Sequence 317 to 366 deleted from MHC-ftz-∆i |
| MHC-ftz-∆i Del 7 | Sequence 367 to 416 deleted from MHC-ftz-∆i |
| MHC-ftz-∆i Del 8 | Sequence 417 to 468 deleted from MHC-ftz-∆i |
| MHC-ftz-∆i-βG-∆i | Sequence -20 to 465 of MHC-ftz-∆i inserted at the HindIII site upstream of βG-∆i |
| RC-MHC-ftz-∆i-βG-∆i | The reverse complement of sequence -20 to 465 of MHC-ftz-∆i inserted at the HindIII site upstream of βG-∆i |
| βG-∆i-βG-∆i | Sequence 1 to 441 of βG-∆i inserted at the HindIII site upstream of βG-∆i |
| B1-βG-∆i | Sequence -44 to 285 of βG-∆i inserted at the KpnI site upstream of βG-∆i |
| B2-βG-∆i | Sequence 61 to 390 of βG-∆i inserted at the KpnI site upstream of βG-∆i |
| B3-βG-∆i | Sequence 166 to 495 of βG-∆i inserted at the KpnI site upstream of βG-∆i |
| MHC-βg-Δi Del 1 | Sequence -44 to 2 deleted from MHC-βg-Δi |
| MHC-βg-Δi Del 2 | Sequence 64 to 120 deleted from MHC-βg-Δi |
| MHC-βg-Δi Del 3 | Sequence 121 to 230 deleted from MHC-βg-Δi |
| MHC-βg-Δi Del 4 | Sequence 231 to 340 deleted from MHC-βg-Δi |
| MHC-βg-Δi Del 5 | Sequence 341 to 450 deleted from MHC-βg-Δi |
| MHC-βg-Δi Del 6 | Sequence 429 to 555 deleted from MHC-βg-Δi |
| βG-ftz intron EJ 1 | Sequence 302 to 448 of c-ftz-i inserted downstream of nucleotide 92 of βG-∆i |
| βG-ftz intron EJ 2 | Sequence 302 to 448 of c-ftz-i inserted downstream of nucleotide 315 of βG-∆i |
| B1-∆i | Sequence 286 to 500 deleted from βg-Δi |
